# Utilization of expert opinion in infectious diseases clinical guidelines—A meta-epidemiological study

**DOI:** 10.1371/journal.pone.0306098

**Published:** 2024-06-27

**Authors:** Blin Nagavci, Lukas Schwingshackl, Ignacio Martin-Loeches, Botond Lakatos

**Affiliations:** 1 Doctoral School of Clinical Medicine, Semmelweis University, Budapest, Hungary; 2 Faculty of Medicine, Institute for Evidence in Medicine, Medical Center-University of Freiburg, University of Freiburg, Freiburg, Germany; 3 Department of Intensive Care Medicine, Multidisciplinary Intensive Care Research Organization (MICRO), Leinster, Dublin, Ireland; 4 Division of Infectology, Department of Hematology and Internal Medicine, Semmelweis University, Budapest, Hungary; 5 South Pest Central Hospital, National Institute of Hematology and Infectious Diseases, Budapest, Hungary; National Defense Medical Center, TAIWAN

## Abstract

**Introduction:**

Expert opinion is widely used in clinical guidelines. No research has ever been conducted investigating the use of expert opinion in international infectious disease guidelines. This study aimed to create an analytical map by describing the prevalence and utilization of expert opinion in infectious disease guidelines and analyzing the methodological aspects of these guidelines.

**Methods:**

In this meta-epidemiological study, systematic searches in PubMed and Trip Medical Database were performed to identify clinical guidelines on infectious diseases, published between January 2018 and May 2023 in English, by international organizations. Data extracted included guideline characteristics, expert opinion utilization, and methodological details. Prevalence and rationale of expert opinion use were analyzed descriptively. Methodological differences between groups were analyzed with Chi-square and Mann-Whitney U Test.

**Results:**

The analysis covered 66 guidelines with 2296 recommendations, published/endorsed by 136 organizations. Most guidelines (79%) used systematic literature searches, 42% provided search strategies, and 38% presented screening flow diagrams and conducted risk of bias assessments. 48.5% of the guidelines allowed expert opinion, most of which included expert opinion as part of the evidence hierarchy within the grading system. Guidelines allowing expert opinion, compared to those which do not, issued more recommendations per guideline (48.82 vs.19.13, p<0.001), and reported fewer screening flow diagrams (25% vs. 65%, p = 0.002), and less risk of bias assessments (19% vs.78%, p<0.001).

**Conclusions:**

Expert opinion is utilized in half of assessed guidelines, often integrated into the evidence hierarchy within the grading system. Its utilization varies considerably in methodology, form, and terminology between guidelines. These findings highlight a pressing need for additional research and guidance, to improve and advance the standardization of infectious disease guidelines.

## Introduction

Clinical practice guidelines are statements with recommendations developed by experts in a particular medical field, guiding healthcare professionals in making informed decisions about patient care [1]. These documents are typically created through a standardized and transparent approach including a systematic review of medical literature, and they usually consider a range of available evidence, including systematic reviews, clinical trials, observational studies such as cohort studies and less often case series or case reports. In some instances, when high-quality evidence is not available, guideline developers might rely on clinical judgement or expert opinion (EO) for issuing recommendations [2–4]. Presently, authors hold diverse definitions and perspectives regarding the concept and use of EO in clinical guidelines. For example, Eibling et al. consider EO not only as personal experience gained over the years but also as knowledge accumulated from a wide range of sources, which should be considered in clinical guidelines [5]. On the other hand, Schünemann et al. consider EO solely an opinion, which needs to be separated from any type of evidence and should not be used as a source for recommendations [6, 7]. Various organizations and societies have different policies and utilize EO in different ways, some allowing it to make recommendations in certain situations [8, 9], while others do not [7, 10]. Nevertheless, EO continues to be used in clinical guidelines. Up to one-quarter of guidelines, published between 2010 to 2016 in different topics, issued recommendations based on EO, 91% of which did not show an explicit rationale for EO use [2]. In critical care guidelines, 10% of strong recommendations were based on EO [11], while in cardiology guidelines up to 55% of recommendations were based on EO [12]. There is a lack of clarity or consensus on how EO should be used in clinical guidelines. Guideline developers employ and present EO in different ways, often without providing a rationale for the recommendations, whether by issuing recommendations based only on EO when there is no evidence available, considering low-quality observational data or indirect data as expert opinion, or by using EO as a level of evidence [2]. This has the potential to impede clinicians’ understanding of the underlying evidence supporting clinical recommendations and might affect guideline implementation [2, 3]. The exact implications of this issue for clinical practice remain uncertain and warrant further investigation.

When it comes to infectious diseases (ID) guidelines, they too are prone to such inconsistencies in evidence interpretation and subjectivity. An extra layer of complexity to synthesize and interpret evidence arises from the unique nature of ID, in which epidemiology, diagnosis and treatment might affect individual populations or exhibit pathogenicity only at certain conditions or geographic locations [13]. Despite the large number of guidelines on ID, and their importance, no research has been conducted that investigates the use of EO in this field of medicine. We postulated that international societies and organizations use diverse approaches and methodologies for utilizing EO.

Bearing in mind the impact of such guidelines, this meta-epidemiological study aimed to create an analytical map of international ID guidelines and EO use, by describing the prevalence and utilization of EO and analyzing the methodological aspects of these guidelines.

## Methods

This meta-epidemiological study is designed and reported in accordance with guidelines for reporting meta-epidemiological research [14]. An internal protocol was used for methodological consistency of this project (S1 Appendix).

### Literature searches

Systematic literature searches were conducted in two main databases, PubMed, and the Trip Medical Database, in May 2023. A combination of Medical Subject Headings (MeSH) terms and keywords was used with a focus on achieving high sensitivity. The searches targeted international infectious disease guidelines published within the last 5.5 years (from January 1, 2018, to May 15, 2023) to ensure the inclusion of most recent guidance. No additional filters were applied. Detailed search strategies can be accessed in the S1 Appendix.

### Identification of relevant guidelines

**Fig 1:**
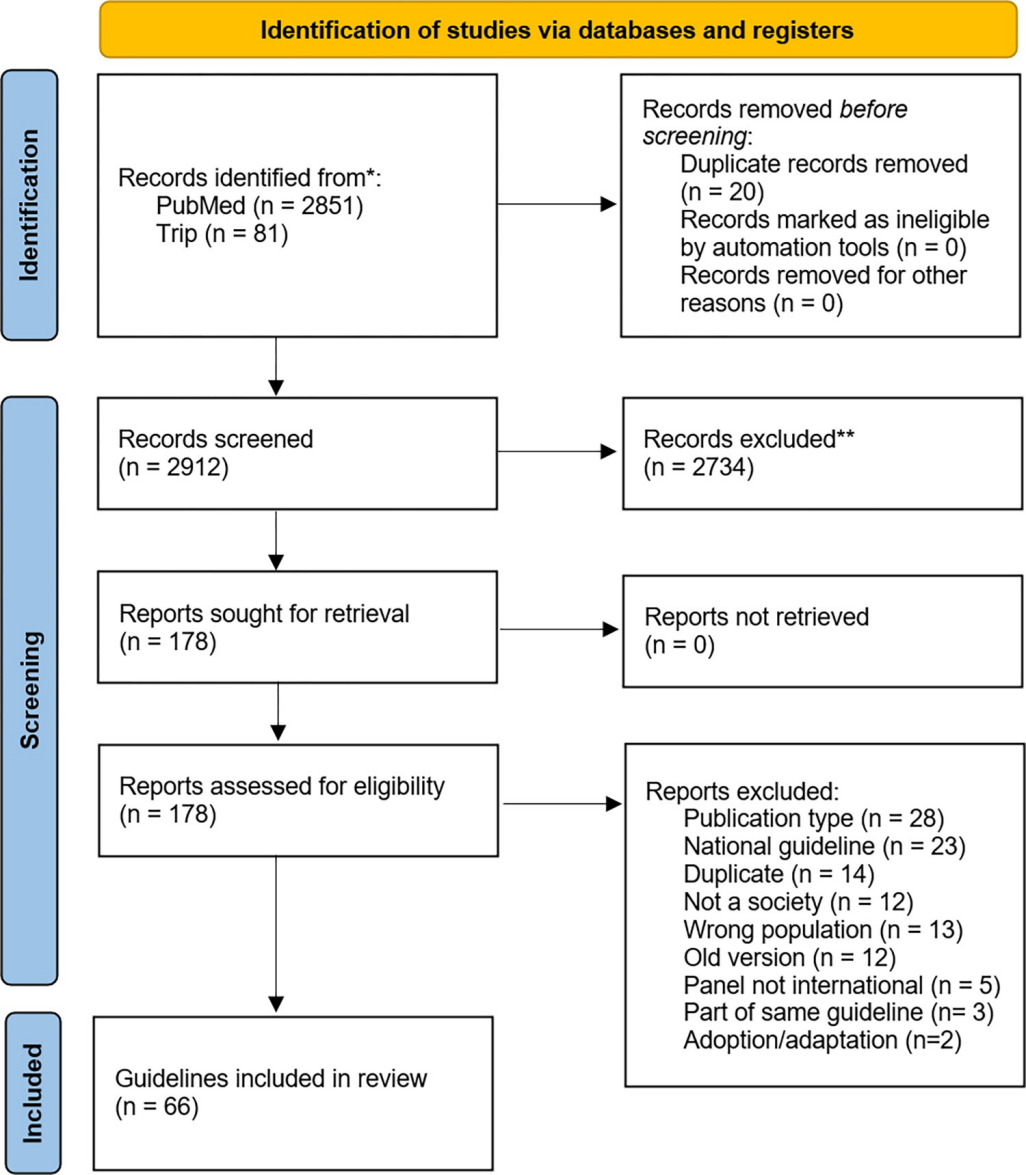
PRISMA flow diagram.

The screening process for relevant guidelines was conducted in two phases: title/abstract screening and full-text screening, using Rayyan (www.rayyan.ai) by a single reviewer (BN). Retrieved guidelines were evaluated and included if they met the following criteria: 1) clinical guidelines pertaining to any infectious disease, 2) published by an international society or organization involving at least two countries, 3) utilized primary studies (e.g. trials, cohort studies etc.) for evidence synthesis, 4) published in the English language, 5) published from 01.01.2018 onwards, and 6) focused on guidelines for humans rather than animals. Conversely, the following documents were excluded: public health guidelines, documents not published by a society/organization, documents authored by individuals from a single country, and guidelines that adopted recommendations from other guidelines. An exception was made for two national societies, namely the National Institute for Health and Care Excellence (NICE) and Infectious Diseases Society of America (IDSA), which were considered relevant for inclusion due to their significant international impact. In cases where the systematic searches yielded multiple versions of a guideline (e.g., living guidelines), only the most recent version was included and assessed. The screening results, along with the reasons for exclusion, are presented using the Preferred Reporting Items for Systematic Reviews and Meta-Analyses (PRISMA) flow diagram [15], in Fig 1.

### Data extraction

The data extraction process was conducted using piloted Excel sheets by one reviewer (BN). The extracted data for each guideline include title, authors, publication year, publishing and endorsing societies, methodological details (systematic searches, risk of bias assessment, evidence appraisal systems, screening flowcharts), details on EO use, terminology used for EO recommendations, and number of recommendations (complete list in the S1 Appendix).

To assess the utilization of EO in the included guidelines, both the methods section and the supplementary materials were thoroughly examined. The evidence grading systems were evaluated to determine if they incorporated EO, and all issued recommendations and their corresponding evidence ratings were assessed for each guideline. In cases where guidelines did not provide sufficient information, additional sources such as society websites and guideline manuals were consulted.

### Data analysis

Descriptive statistics were used for assessing the prevalence of EO use. The definitions and rationale of the EO use in the included guidelines were presented narratively. Distribution of data was assessed with the Shapiro–Wilk test for normality. Chi-square and Mann-Whitney U Test were used to compare methodological differences between groups, with an α level 0.05. ChatGPT was used for grammar corrections and readability. Upon utilizing this tool, the authors made necessary edits to the content, assuming full responsibility for the publication’s accuracy and integrity.

## Results

### Search results

The systematic literature searches yielded a total of 2,912 references, which were initially screened based on their title and abstract, and 178 were selected for full-text assessment. Finally, a total of 66 guidelines were deemed relevant and were included in this study (Fig 1). Included guidelines were published and endorsed by 136 distinct societies or organizations (S1 Appendix). The cumulative number of recommendations provided across all included guidelines was 2296.

### Methodological characteristics

Among the 66 included guidelines, 79% of them reported use of systematic literature searches, and 67% explicitly mentioned the electronic databases that were searched, while 42% provided full details of their search strategies. 38% presented screening flow diagrams (e.g., PRISMA or similar) to depict the screening process, and the same percentage conducted a risk of bias assessment. The decision-making processes for issuing recommendations was detailed in 68% of guidelines (Table 1).

**Table 1:** Characteristics of included guidelines.

**General characteristics of included guidelines (n = 66)**	**Number**
Publishing and endorsing societies	136
Included guidelines	66
Total number of recommendations*	2296
Mean rate of recommendations per guideline, mean (sd)	35.9 (31.9)
**Methodological details**	**Number of guidelines (%)**
Literature searches reported	52 (78.8%)
Electronic databases presented	44 (66.7%)
Search strategies presented	28 (42.4%)
PRISMA flowcharts (or similar) presented	25 (37.9%)
Risk of bias assessment reported	25 (37.9%)
System to assess QoE reported	62 (93.9%)
System to assess SoR reported	62 (93.9%)
Decision making process explained	45 (68.2%)
EO use reported/allowed	32 (48.5%)
EO use not reported	23 (34.8%)
EO use unknown†	11 (16.7%)
**Number of recommendations in guidelines allowing EO (n = 32)**	**Number (%)**
Number of recommendations*	1465 (100%)
Number of recommendations based on expert opinion or on a level of evidence where expert opinion is part of	448 (30%)

PRISMA: The Preferred Reporting Items for Systematic reviews and Meta-Analyses, EO: Expert Opinion, QoE: Quality of Evidence, SoR: Strength of Recommendations.

*For two guidelines, the number of recommendations could not be calculated as they were not marked or delineated.

†Not possible to assess, due to lack of a methods section or lack of presenting the QoE and SoR. They were excluded from analyses.

The use of a grading system for evaluation of evidence as well as a rating of the strength of recommendations was carried out by 94% of the included guidelines. The most used system was the Grading of Recommendations Assessment, Development, and Evaluation (GRADE) with 47% [7], followed by the Infectious Diseases Society of America-US Public Health Service System (IDSA-USPHS) with 16% [16] (S1 Appendix). In 14% of the guidelines, a system could not be identified due to lack of detailed information.

### Utilization of expert opinion

Among the 66 guidelines included in the study, 32 (48.5%) reported that they allowed or utilized EO in formulating recommendations. In contrast, 23 (34.8%) did not report the use or utilization of EO, while 11 (16.6%) of guidelines did not provide sufficient information to assess whether EO was allowed or not, mostly due to the lack of a methods section or lack of presenting the Quality of Evidence (QoE) in the recommendations.

In the 32 guidelines that incorporated EO, a total of 1,465 recommendations were issued, of which 448 (30%) were classified as either recommendations based on EO or based on evidence levels that contained EO.

**Fig 2:**
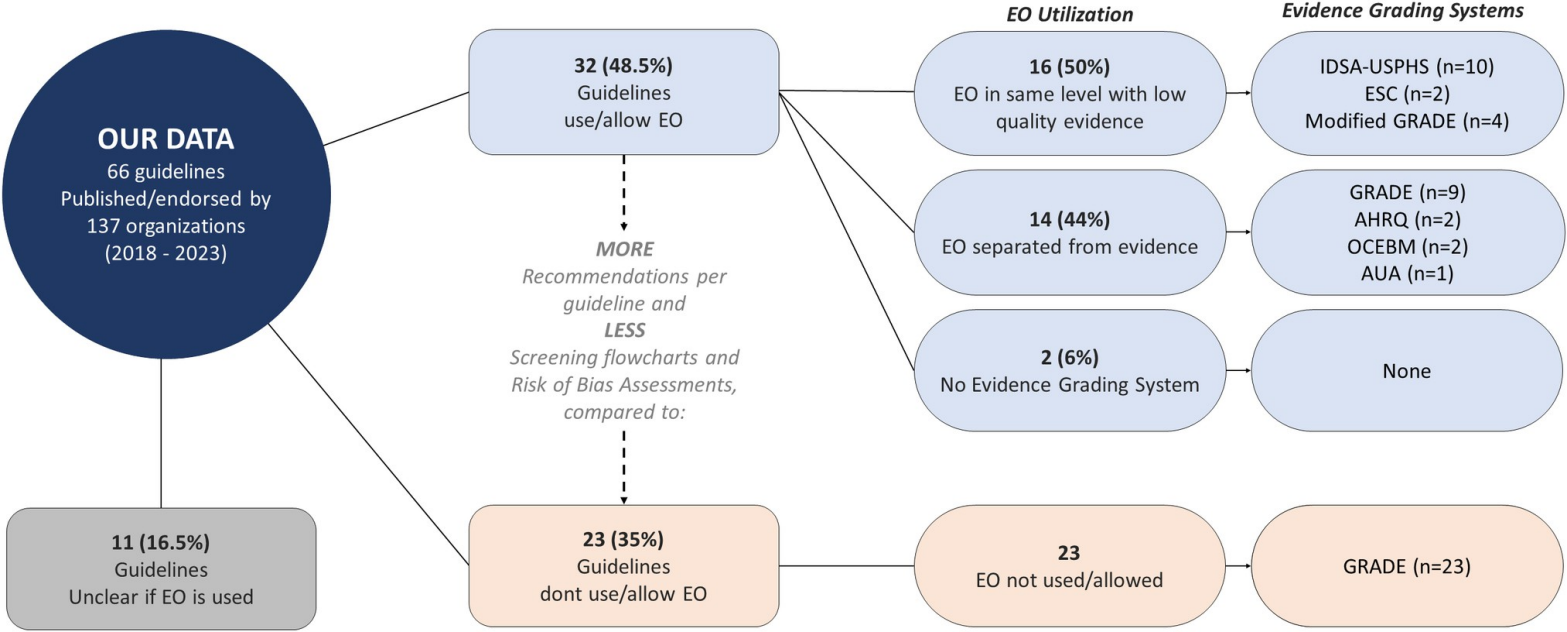
Visual description of EO utilization and evidence grading systems. EO: Expert Opinion, IDSA-USPHS: The Infectious Diseases Society of America-US Public Health Service, ESC: European Society of Cardiology, GRADE: Grading of Recommendations, Assessment, Development, and Evaluations, AHRQ: Agency for Healthcare Research and Quality, OCEBM: Oxford Centre for Evidence-Based Medicine, AUA: American Urological Association.

Within the subset of these 32 guidelines, 16 used evidence grading systems in which EO is allowed and listed within the same evidence level as low or very low-quality evidence (Fig 2). For example: *“Level of evidence C*: *Consensus of opinion of the experts and/or small studies*, *retrospective studies*, *registries”* [17], or *“Quality of Evidence III*: *Evidence from opinions of respected authorities*, *based on clinical experiences*, *descriptive studies*, *or reports of expert committees”* [18]. The evidence grading systems used in this group of guidelines were: The Infectious Diseases Society of America-US Public Health Service (IDSA-USPHS) system [18], European Society of Cardiology (ESC) system [17] and systems modified from GRADE (Fig 2). From this group, only one guideline delineated EO from very low/low-quality evidence, by explaining this in each recommendation [19].

On the other hand, 14 guidelines used evidence grading systems where EO is not incorporated in the hierarchy of evidence, or it is categorized separately from other types of evidence. Nine of these guidelines followed the Grading of Recommendations, Assessment, Development, and Evaluations (GRADE) approach [7], which does not incorporate EO, though EO was still used in these guidelines. Five guidelines adhered to the Agency for Healthcare Research and Quality (AHRQ) [20], Oxford Centre for Evidence-Based Medicine (OCEBM) [21], or American Urological Association (AUA) systems [22], where EO is isolated from other types of evidence and has its own distinct level. Two guidelines did not use evidence grading systems. Details available in Fig 2.

From the 23 guidelines which did not report to have utilized EO, all used the GRADE approach for assessing the quality of evidence [7]. The complete list with assessed guidelines and respective evidence grading systems can be found in the S1 Appendix.

### Methodological differences

Significant differences in methodology were observed between guidelines which allowed EO and those that did not. In the group that allowed EO, on average, a higher number of recommendations were issued per guideline compared to the group that did not allow EO (48.8 vs. 19.1, p<0.001). Additionally, a smaller proportion of guidelines in the EO group provided screening flow diagrams (25% vs. 65%, p = 0.002), and fewer guidelines reported conducting risk of bias assessments (19% vs. 78%, p<0.001) (Table 2). No significant differences were observed for literature searches, electronic databases, search strategy reporting and presence of systems for evidence appraisal (Table 2).

**Table 2:** Methodological comparisons of guidelines allowing EO and guidelines not allowing EO.

	Guidelines allowing EO	Guidelines not allowing EO	Guidelines EO status unclear*	p value†
	(n = 32)	(n = 23)	(n = 11)	
Total recommendations Number (min/max)	1465 (3/153)	440 (2/57)	391 (9/125)	/
Mean number of recommendations per guideline Mean (sd)	48.82 (35.49)	19.13 (14.93)	65.16 (33.54)	0.000^‡^
Literature searches reported Number (%)	29 (91%)	21 (91%)	2 (18%)	0.931
Electronic databases presented Number (%)	24 (75%)	18 (78%)	2 (18%)	0.778
Search strategy reported Number (%)	13 (41%)	14 (61%)	1 (9%)	0.138
PRISMA (or similar) presented Number (%)	8 (25%)	15 (65%)	1 (9%)	0.002
Risk of bias assessment reported Number (%)	6 (19%)	18 (78%)	2 (18%)	0.000
System to assess QoE and SoR reported Number (%)	30 (94%)	23 (100%)	9 (9%)	NA^§^
Decision making process explained Number (%)	24 (75%)	21 (91%)	0 (0%)	0.122

EO: Expert opinion, QoE: Quality of evidence, SoR: Strength of recommendations.

*Not possible to assess if EO is allowed or not, mostly due to missing methods section. Most of them (n = 9) were published by American Society of Transplantation. This group has been excluded from analyses.

† Chi-square test, comparing Guidelines allowing EO with guidelines not allowing EO.

‡ Mann-Whitney U Test

§ Not possible to calculate due to low number of events.

### Terminology and phrasing

In the 32 guidelines allowing EO, eight different terms were identified to label EO as a concept. The most prevalent terms were "Opinions of respected authorities," "Expert opinion," "Expert judgment," and "Consensus of expert opinion." Followed by "Consensus recommendation," "Good practice principle," and "In our practice statement" (S1 Appendix).

A diverse range of terminology was observed regarding the phrasing of EO-based recommendations. A total of 42 main verbs were identified to describe the 448 recommendations based on EO (or in evidence level containing EO). The most used verbs were "should," "recommend," "consider," "may," "is," "indicated," and "suggest." The complete list of verbs can be found in S1 Appendix.

## Discussion

### Summary of findings

Our results showed that half of the included guidelines allowed or used EO for formulating recommendations, with one third of their total number of recommendations being based on EO, or within an evidence level where EO is listed. In most cases, EO was incorporated into the evidence hierarchy and placed within the same category as low or very low-quality evidence. The phrasing and presentation of EO recommendations varied across guidelines in structure and form. Guidelines allowing EO issued more recommendations on average and reported less frequent use of screening flow diagrams and risk of bias assessments.

### Comparisons with similar studies

In a meta-epidemiological study conducted by Ponce et al. [2], 69 guidelines from various fields, predominantly in endocrinology, were examined to identify the rationale behind EO recommendations. Relatively similar to our findings, 37.9% of recommendations in their sample had a level of evidence designated as EO. However, it is important to note that all guidelines in their sample were based on systematic reviews, whereas in our study, less than 80% utilized literature searches. This disparity may be attributed to methodological differences between the two studies. Ponce et al. specifically focused on guidelines from various fields and excluded those that did not employ EO, while our study focused on ID and included a more representative sample, as EO use was not an inclusion criterion but an outcome.

A study published by Mitchel et al. [23], examined 31 infection prevention and control guidelines revealing that 41.5% of recommendations were based on evidence from descriptive studies, EO, and low-quality evidence. While there are similarities between their findings and ours, our study focuses on clinical guidelines and not on infection prevention and control. Also, our methodology differs as we employed a systematic approach.

### Implications

This study identified several challenges with current guidelines and their development processes. First, one-fifth of the guidelines did not utilize literature searches, indicating a lack of a systematic approach in identifying and synthesizing evidence. In addition, only half of the guidelines provided search strategies, suggesting a gap in transparency and reproducibility. Furthermore, less than half of the guidelines provided a flow diagram or risk of bias assessments, indicating a potential lack of methodological rigor in evidence synthesis. These findings highlight the need for more adherence to methodological principles for developing trustworthy guidelines. Similar results were reported also by previous studies [24–26]. On the other hand, it was encouraging to see that nearly all guidelines used an evidence-grading system.

Second, half of the included guidelines allowed EO to formulate their recommendations, with 30% of their overall number of recommendations being based on EO or in an evidence level where EO is listed. Most of these guidelines used evidence grading systems where EO is part of the evidence hierarchy, placing EO in the same category as low or very low-quality evidence. This lack of distinction between EO and other types of evidence in the recommendations might pose a challenge [27]. For readers, it might be unclear whether a recommendation is based solely on clinical experience, or on very low-quality evidence such as case reports and case series, or on large observational studies, which might provide more solid evidence for basing recommendations. This lack of distinction between EO and evidence may result in misunderstandings regarding the strength and reliability of recommendations among clinicians, therefore, efforts need to be taken to make this clear in each recommendation where EO is used.

Third, there were statistically significant differences between guidelines that allowed EO and those that did not. The former issued a higher number of recommendations on average and reported less frequent use of screening flow diagrams and risk of bias assessments. As this study provides only a “map”, further research is necessary to explore this issue for a more detailed understanding of these differences.

Fourth, our findings highlight a major diversity in the terminology used to express recommendations. Especially regarding their strength and the verbs employed, both within and across different guidelines. An illustrative instance includes the shared use of the term "we recommend". This term is used for recommendations based on high-quality evidence and also for recommendations stemming entirely from EO. While we acknowledge the methodological differences among various societies and organizations, it’s imperative to consider the perspective of end users responsible for patient care. This array of terminology can potentially lead to confusion in the practical application of such recommendations. More research is needed in this field, and more efforts need to be made towards standardization of wording among international guidelines.

### Strengths and limitations

This study has several strengths. It is the first study providing an analytical map of EO utilization in international clinical guidelines on ID and analyzing methodological aspects and differences. The study fills a critical gap in the current literature by shedding light on the patterns, prevalence, and characteristics of EO use in the ID guideline landscape. By employing systematic search strategies, a comprehensive collection of ID guidelines, from 136 distinct organizations, was identified, ensuring a representative sample for analysis. By providing an analytical map of EO utilization in ID guidelines, this study generates insights into current practices and patterns, which can guide the development of future guidance documents and regulatory frameworks to ensure standardized, transparent, and reliable use of EO or clinical experience in clinical guidelines.

This study has a few limitations. One is the exclusive assessment of guidelines published in English language, which may limit the generalizability of the findings to guidelines in other languages. However, since the study’s primary focus was on international guidelines, which are typically published in English, we do not consider this a major limitation. This is supported by the lack of identified international guidelines in languages other than English. Another potential limitation is that the screening and data extraction were performed by a single reviewer. To minimize any possible errors, we utilized a detailed protocol with clear inclusion criteria (S1 Appendix) and piloted data extraction sheets. Additionally, S1 Appendix provides a comprehensive list of all excluded guidelines and their respective exclusion reasons, ensuring maximal transparency.

## Conclusions

Half of international infectious disease guidelines use or allow expert opinion. In most cases, expert opinion is part of evidence hierarchy within the evidence grading systems. Its utilization varies considerably in methodology, form, and terminology between guidelines. These findings highlight a pressing need for more guidance and standardization in infectious disease guidelines.

## Supporting information

S1 Checklist PRISMA 2020 checklist. (DOCX)

S1 Appendix (DOCX)
