# Supplementary material for: Utilization of expert opinion in infectious diseases clinical guidelines—A meta-epidemiological study
Source: PLoS One. 2024 Jun 27;19(6):e0306098. doi: 10.1371/journal.pone.0306098 (PMC11210760; doi:10.1371/journal.pone.0306098)
Supplement: S1 Appendix — (DOCX) [file pone.0306098.s002.docx]

S1 Appendix

**Utilization of Expert Opinion in Infectious Diseases Clinical Guidelines - A Meta-Epidemiological Study**

**Blin Nagavci^1*^, Lukas Schwingshackl^2^, Ignacio Martin-Loeches^3^, Botond Lakatos^4,5^**

*1) Doctoral School of Clinical Medicine, Semmelweis University, Budapest, Hungary.*

*2) Institute for Evidence in Medicine, Medical Center-University of Freiburg, Faculty of Medicine, University of*

*Freiburg, Freiburg, Germany*

*3) Department of Intensive Care Medicine, Multidisciplinary Intensive Care Research Organization (MICRO), Leinster D08NYH1, Dublin, Ireland.*

*4) Division of Infectology, Department of Hematology and Internal Medicine, Semmelweis University, Budapest, Hungary.*

*5) South Pest Central Hospital, National Institute of Hematology and Infectious Diseases, Budapest, Hungary.*

*Corresponding author

Email: dr.bnagavci@gmail.com (BN)

Contents

[Protocol 3](#_Toc169176141)

[Search strategies 5](#_Toc169176142)

[S1 Table: List of included guidelines. 6](#_Toc169176143)

[S2 Table: Type of systems used for assessing quality of evidence in guidelines reporting use of a system. 17](#_Toc169176144)

[S3 Table: Terms used for EO 18](#_Toc169176145)

[S4 Table: List of verbs used for phrasing EO recommendations. 19](#_Toc169176146)

[S5 Table: Publishing and endorsing organizations of included guidelines. 21](#_Toc169176147)

[S6 Table: Excluded guidelines during full-text screening, with reasons. 27](#_Toc169176148)

## Protocol

**Title**

Utilization of Expert Opinion in Infectious Diseases Clinical Guidelines - A Meta-Epidemiological Study

**Background and Objectives**

Clinical practice guidelines, crucial for informed healthcare decisions, often rely on expert opinion (EO) when robust evidence is lacking. Perspectives on EO vary, some considering it a blend of experience and diverse knowledge, while others see it as a standalone opinion without evidentiary support. The utilization of EO in guidelines varies among organizations, leading to inconsistencies. In certain medical fields, up to 25% of guidelines issued recommendations based on EO. In infectious diseases (ID) guidelines, the complex nature of ID introduces further challenges, with epidemiological variations influencing recommendations. Despite the significance of ID guidelines, there is a notable absence of research on EO use in this area. This meta-epidemiological study seeks to address this gap, aiming to provide insights into the prevalence, utilization, and methodological aspects of EO in international ID guidelines.

**Information sources**

- PubMed (<https://pubmed.ncbi.nlm.nih.gov/>)
- Trip Medical Database (<https://www.tripdatabase.com/>)

Complete search strategies will be presented in the supplementary material.

**Selection process**

The screening process for relevant guidelines will be conducted in two phases: title/abstract screening and full-text screening, using Rayan (www.rayyan.ai), by a single reviewer.

Retrieved guidelines will be evaluated and included if they meet the inclusion criteria.

**Eligibility criteria**

- Clinical guidelines in ID (exclude public health guidelines, management etc.)
- Any topic/infection within ID.
- Clinical guidelines published by an international society or an international organization (at least two countries).
- Clinical guidelines published by a group of authors from at least two different countries.
- Clinical guidelines using primary studies as a source. Guidelines adopting recommendations from other guidelines will be excluded, as it is not possible to assess EO.
- Clinical guidelines published in English language.
- Clinical guidelines published in the period 2018-2023.
- NICE and IDSA clinical guidelines will be considered, due to their international usage.
- If the search captured several updates of the same guideline, only the latest version will be considered.

**Data collection process**

The extraction process will be conducted in piloted Excel sheets, by a single reviewer.

**Data items**

The following items for each relevant guideline will be extracted:

- Title
- year
- Journal
- Authors
- DOI/Link
- Publishing organizations
- Number of publishing organizations
- Literature searches reported
- Databases reported
- Full search string presented
- PRISMA or similar presented
- RoB assessment
- System to assess QoE
- System for Strength of Recommendations
- Decision making/agreement process explained in methods
- Recommendations clearly presented and delineated from text
- EO utilization
- The term for describing EO (e.g. expert opinion, best practice)
- EO recommendations (or level where EO is) are clearly phrased/delineated from other recommendations
- Verbs/terms used to present recommendations
- Total number recommendations
- Total number of EO recommendations

**Risk of bias Assessment**

Risk of bias assessment is not relevant for this study and will not be assessed.

**Data analysis**

Descriptive statistics will be employed to assess the prevalence of EO use. The definitions and rationale for EO use in the included guidelines will be presented in a narrative format. The distribution of data will be evaluated using the Shapiro–Wilk test for normality. Methodological differences between groups will be compared using the Chi-square (for proportions) and Student's t-test (for means), with a significance level set at α = 0.05. If data is not normally distributed Mann-Whitney U Test will be used to compare means.

## Search strategies

**PubMed** (<https://pubmed.ncbi.nlm.nih.gov/>)

Search date: 15.05.2023

| Search | Query | Results |
| --- | --- | --- |
| #5 | Search: #1 AND #2 Filters: English, from 2018 - 2023 Sort by: Most Recent | 2,851 |
| #4 | Search: #1 AND #2 Filters: from 2018 - 2023 Sort by: Most Recent | 3,108 |
| #3 | Search: #1 AND #2 Sort by: Most Recent | 10,584 |
| #2 | Search: (guideline[Title]) OR (guideline*[Title]) Sort by: Most Recent | 93,403 |
| #1 | Search: (("Infections"[Mesh]) OR (infection[Title])) OR (infectious[Title]) Sort by: Most Recent | 3,140,812 |

**Trip Medical Database** (<https://www.tripdatabase.com/>)

Search date: 15.05.2023

| Any of these words (title) | Infections, infection, infectious |
| --- | --- |
| This exact phrase (title) | guideline |
| Timeframe | 2018-2023. |
| Results | 324 |
| Document type: Guidelines | 81 |

## S1 Table: List of included guidelines.

| ***No*** | ***Title*** | | ***Topic/Infection*** | ***Year*** | ***Doi*** | ***Publishing society*** | ***System to assess quality of Evidence (QoE)*** | ***System to rate strength of recommendations (SoR)*** | ***EO use reported/***  ***allowed*** |
| --- | --- | --- | --- | --- | --- | --- | --- | --- | --- |
| 1 | 2018 Infectious Diseases Society of America Clinical Practice Guideline for the Management of Outpatient Parenteral Antimicrobial Therapy | | Antimicrobial Therapy | 2019 | doi:10.1093/cid/ciy867 | Infectious Diseases Society of America (IDSA) | GRADE | GRADE | no |
| 2 | 2019 update of the European AIDS Clinical Society Guidelines for treatment of people living with HIV version 10.0 | | HIV | 2020 | doi:10.1111/hiv.12878 | European AIDS Clinical Society (EACS) | none | none | yes |
| 3 | 2019 update of the WSES guidelines for management of Clostridioides (Clostridium) difficile infection in surgical patients | | Clostridium difficile | 2019 | doi: 10.1186/s13017-019-0228-3 | World Society of Emergency Surgery | Modified GRADE | Modified GRADE | no |
| 4 | 2020 European guideline on the management of genital molluscum contagiosum | | Genital molluscum contagiosum | 2021 | doi:10.1111/jdv.16856 | International Union Against Sexually Transmitted Infections (IUSTI), European Dermatology Forum (EDF), Dermato-venereological Branch of the European Union of Medical Specialists (UEMS), European Academy of Dermatology and Venereology (EADV), | Modified GRADE | Modified GRADE | yes |
| 5 | 2020 European guideline on the management of syphilis | | Syphilis | 2021 | doi:10.1111/jdv.16946 | International Union Against Sexually Transmitted Infections (IUSTI) | Modified GRADE | Modified GRADE | yes |
| 6 | 2021 European guideline on HIV testing in genito-urinary medicine settings | | HIV | 2021 | doi:10.1111/jdv.17139 | International Union Against Sexually Transmitted Infections (IUSTI) | Modified GRADE | Modified GRADE | yes |
| 7 | 2021 European guideline on the management of Mycoplasma genitalium infections | | Mycoplasma genitalium | 2022 | doi:10.1111/jdv.17972 | International Union Against Sexually Transmitted Infections (IUSTI) | Modified GRADE | Modified GRADE | yes |
| 8 | 2021 European Guideline on the management of proctitis, proctocolitis and enteritis caused by sexually transmissible pathogens | | Proctitis, proctocolitis and enteritis | 2021 | doi:10.1111/jdv.17269 | International Union Against Sexually Transmitted Infections (IUSTI) | AHRQ | AHRQ | yes |
| 9 | 8th European Conference on Infections in Leukaemia: 2020 guidelines for the diagnosis, prevention, and treatment of invasive fungal diseases in paediatric patients with cancer or post-haematopoietic cell transplantation | | Invasive fungal diseases | 2021 | doi:10.1016/s1470-2045(20)30723-3 | European Conference on Infections in Leukaemia (ECIL) | IDSA-USPHS | Modified IDSA-USPHS | yes |
| 10 | APSIC guidelines for the prevention of surgical site infections | | Surgical site infections | 2019 | doi:10.1186/s13756-019-0638-8 | The Asia Pacific Society of Infection Control (APSIC) | IDSA-USPHS | IDSA-USPHS | yes |
| 11 | Asian Pacific association for the study of liver (APASL) guidelines: hepatitis B virus in pregnancy | | Hepatitis B virus | 2022 | doi:10.1007/s12072-021-10285-5 | Asian Pacific association for the study of liver (APASL) | Modified GRADE | Modified GRADE | no |
| 12 | Clinical Practice Guideline by the Infectious Diseases Society of America (IDSA) and Society for Healthcare Epidemiology of America (SHEA): 2021 Focused Update Guidelines on Management of Clostridioides difficile Infection in Adults | | Clostridioides difficile | 2021 | doi:10.1093/cid/ciab549 | Infectious Diseases Society of America (IDSA), Society for Healthcare Epidemiology of America (SHEA) | GRADE | GRADE | no |
| 13 | Clinical Practice Guideline by the Pediatric Infectious Diseases Society and the Infectious Diseases Society of America: 2021 Guideline on Diagnosis and Management of Acute Hematogenous Osteomyelitis in Pediatrics | | Hematogenous Osteomyelitis | 2021 | doi:10.1093/jpids/piab027 | Infectious Diseases Society of America (IDSA) | GRADE | GRADE | no |
| 14 | Clinical Practice Guideline for the Management of Asymptomatic Bacteriuria: 2019 Update by the Infectious Diseases Society of America | | Asymptomatic bacteriuria | 2019 | doi:10.1093/cid/ciz021 | Infectious Diseases Society of America (IDSA) | GRADE | GRADE | no |
| 15 | Clinical Practice Guidelines by the Infectious Diseases Society of America (IDSA), American Academy of Neurology (AAN), and American College of Rheumatology (ACR): 2020 Guidelines for the Prevention, Diagnosis, and Treatment of Lyme Disease | | Lyme Disease | 2021 | doi:10.1002/art.41562 | Infectious Diseases Society of America (IDSA), American Academy of Neurology (AAN), and American College of Rheumatology (ACR) | GRADE | GRADE | no |
| 16 | Clinical Practice Guidelines by the Infectious Diseases Society of America (IDSA): 2020 Guideline on Diagnosis and Management of Babesiosis | | Babesiosis | 2021 | doi:10.1093/cid/ciab050 | Infectious Diseases Society of America (IDSA) | GRADE | GRADE | no |
| 17 | Clinical Practice Guidelines by the Infectious Diseases Society of America: 2018 Update on Diagnosis, Treatment, Chemoprophylaxis, and Institutional Outbreak Management of Seasonal Influenza | | Seasonal influenza | 2019 | doi:10.1093/cid/ciy874 | Infectious Diseases Society of America (IDSA) | IDSA-USPHS | IDSA-USPHS | yes |
| 18 | Consensus guidelines for the diagnosis and management of invasive candidiasis in haematology, oncology and intensive care settings, 2021 | | Candidiasis | 2021 | doi:10.1111/imj.15589 | Australasian Antifungal Guidelines Steering Committee | IDSA-USPHS | Modified GRADE | yes |
| 19 | COVID-19 rapid guideline: managing the long-term effects of COVID-19 | | COVID-19 | 2022 | Website | National Institute for Health and Care Excellence (NICE), Scottish Intercollegiate Guidelines Network (SIGN) and Royal College of General Practitioners (RCGP) | GRADE | GRADE | yes |
| 20 | Cryptococcosis in solid organ transplantation-Guidelines from the American Society of Transplantation Infectious Diseases Community of Practice | | Cryptococcosis | 2019 | doi:10.1111/ctr.13543 | American Society of Transplantation (ASL) | unclear | unclear | unclear* |
| 21 | Cytomegalovirus in solid organ transplant recipients-Guidelines of the American Society of Transplantation Infectious Diseases Community of Practice | | Cytomegalovirus | 2019 | doi:10.1111/ctr.13512 | American Society of Transplantation (ASL) | unclear | unclear | unclear* |
| 22 | Diagnosis and management of patients with Lyme disease: NICE guideline | | Lyme disease | 2018 | doi:10.3399/bjgp18X699713 | National Institute for Health and Care Excellence (NICE) | GRADE | Modified GRADE | yes |
| 23 | Diagnosis and Treatment of Adults with Community-acquired Pneumonia. An Official Clinical Practice Guideline of the American Thoracic Society and Infectious Diseases Society of America | | Community-acquired Pneumonia | 2019 | doi:10.1164/rccm.201908-1581ST | American Thoracic Society (ATS)m Infectious Diseases Society of America (IDSA) | GRADE | GRADE | no |
| 24 | Diagnosis and Treatment of Neurocysticercosis: 2017 Clinical Practice Guidelines by the Infectious Diseases Society of America (IDSA) and the American Society of Tropical Medicine and Hygiene (ASTMH) | | Neurocysticercosis | 2018 | doi:10.4269/ajtmh.18-88751 | Infectious Diseases Society of America (IDSA), American Society of Tropical Medicine and Hygiene (ASTMH) | GRADE | GRADE | yes |
| 25 | EASL Clinical Practice Guidelines on hepatitis E virus infection | | Hepatitis E | 2018 | doi:10.1016/j.jhep.2018.03.005 | European Association for the Study of the Liver (EASL) | Modified GRADE | Modified GRADE | yes |
| 26 | ERS/ESICM/ESCMID/ALAT guidelines for the management of severe community-acquired pneumonia | | Severe community-acquired pneumonia | 2023 | doi:10.1183/13993003.00735-2022 | European Respiratory Society (ERS), European Society of Intensive Care Medicine (ESICM), European Society of Clinical Microbiology and Infectious Diseases (ESCMID), and Latin American Thoracic Association (ALAT) | GRADE | GRADE | no |
| 27 | ESCMID rapid guidelines for assessment and management of long COVID | | COVID-19 | 2022 | doi:10.1016/j.cmi.2022.02.018 | European Society of Clinical Microbiology and Infectious Diseases (ESCMID) | none | none | unclear* |
| 28 | ESCMID/EUCIC clinical practice guidelines on perioperative antibiotic prophylaxis in patients colonized by multidrug-resistant Gram-negative bacteria before surgery | | Antibiotic prophylaxis | 2023 | doi:10.1016/j.cmi.2022.12.012 | European Society of Clinical Microbiology and Infectious Diseases (ESCMID) | GRADE | GRADE | yes |
| 29 | ESCMID-EUCIC clinical guidelines on decolonization of multidrug-resistant Gram-negative bacteria carriers | | Decolonization of multidrug-resistant Gram-negative bacteria | 2019 | doi:10.1016/j.cmi.2019.01.005 | European Society of Clinical Microbiology and Infectious Diseases (ESCMID) | GRADE | GRADE | no |
| 30 | European guidelines for primary antifungal prophylaxis in adult haematology patients: summary of the updated recommendations from the European Conference on Infections in Leukaemia | | Primary antifungal prophylaxis | 2018 | doi:10.1093/jac/dky286 | European Conference on Infections in Leukaemia (ECIL) | IDSA-USPHS | IDSA-USPHS | yes |
| 31 | European Society for Vascular Surgery (ESVS) 2020 Clinical Practice Guidelines on the Management of Vascular Graft and Endograft Infections | | Vascular Graft and Endograft Infections | 2020 | doi: 10.1016/j.ejvs.2019.10.016 | European Society for Vascular Surgery | ESC | ESC | yes |
| 32 | European Society of Clinical Microbiology and Infectious Diseases (ESCMID) guidelines for the treatment of infections caused by multidrug-resistant Gram-negative bacilli (endorsed by European society of intensive care medicine) | | Multidrug-resistant Gram-negative bacilli | 2022 | doi:10.1016/j.cmi.2021.11.025 | European Society of Clinical Microbiology and Infectious Diseases (ESCMID), European society of intensive care medicine (ESICM) | GRADE | GRADE | yes |
| 33 | Evidence-based guideline of the European Association of Nuclear Medicine (EANM) on imaging infection in vascular grafts | | Infection in vascular grafts | 2022 | doi:10.1007/s00259-022-05769-x | European Association of Nuclear Medicine (EANM) | OCEBM | OCEBM | yes |
| 34 | Global guideline for the diagnosis and management of mucormycosis: an initiative of the European Confederation of Medical Mycology in cooperation with the Mycoses Study Group Education and Research Consortium | | Mucormycosis | 2019 | doi:10.1016/s1473-3099(19)30312-3 | European Confederation of Medical Mycology (ECMM), Mycoses Study Group Education & Research Consortium (MSG ERC) | IDSA-USPHS | Modified IDSA-USPHS | yes |
| 35 | Global guideline for the diagnosis and management of rare yeast infections: an initiative of the ECMM in cooperation with ISHAM and ASM | | Yeast infections | 2021 | doi:10.1016/s1473-3099(21)00203-6 | European Confederation for Medical Mycology (ECMM), International Society for Human and Animal Mycology (ISHAM), American Society for Microbiology (ASM) | IDSA-USPHS | Modified IDSA-USPHS | yes |
| 36 | Global guideline for the diagnosis and management of the endemic mycoses: an initiative of the European Confederation of Medical Mycology in cooperation with the International Society for Human and Animal Mycology | | Endemic mycoses | 2021 | doi:10.1016/s1473-3099(21)00191-2 | European Confederation of Medical Mycology (ECMM), International Society for Human and Animal Mycology (ISHAM) | IDSA-USPHS | Modified IDSA-USPHS | yes |
| 37 | Guidelines for the diagnosis, prevention and management of cryptococcal disease in HIV-infected adults, adolescents and children | | Cryptococcal disease | 2018 | Website | World Health Organization (WHO) | GRADE | GRADE | yes |
| 38 | Human parvovirus B19 in solid organ transplantation: Guidelines from the American society of transplantation infectious diseases community of practice | | Parvovirus B19 | 2019 | doi:10.1111/ctr.13535 | American Society of Transplantation (ASL) | unclear | unclear | unclear* |
| 39 | Infectious complications of targeted drugs and biotherapies in acute leukemia. Clinical practice guidelines by the European Conference on Infections in Leukemia (ECIL), a joint venture of the European Group for Blood and Marrow Transplantation (EBMT), the European Organization for Research and Treatment of Cancer (EORTC), the International Immunocompromised Host Society (ICHS) and the European Leukemia Net (ELN) | | Infectious complications | 2022 | doi:10.1038/s41375-022-01570-9 | European Conference on Infections in Leukemia (ECIL), European Group for Blood and Marrow Transplantation (EBMT), the European Organization for Research and Treatment of Cancer (EORTC), the International Immunocompromised Host Society (ICHS) and the European Leukemia Net (ELN) | IDSA-USPHS | Modified IDSA-USPHS | yes |
| 40 | Infectious Diseases Society of America Guidelines on Infection Prevention for Health Care Personnel Caring for Patients with Suspected or Known COVID-19 | | COVID-19 | 2020 | doi: 10.1093/cid/ciaa1063 | Infectious Diseases Society of America (IDSA) | GRADE | GRADE | no |
| 41 | Infectious Diseases Society of America Guidelines on the Diagnosis of COVID-19 | | COVID-19 | 2020 | doi:10.1093/cid/ciaa760 | Infectious Diseases Society of America (IDSA) | GRADE | GRADE | no |
| 42 | Infectious Diseases Society of America Guidelines on the Treatment and Management of Patients with COVID-19 | | COVID-19 | 2023 | Website | Infectious Diseases Society of America (IDSA) | GRADE | GRADE | no |
| 43 | International Society of Cardiovascular Infectious Diseases Guidelines for the Diagnosis, Treatment and Prevention of Disseminated Mycobacterium chimaera Infection Following Cardiac Surgery with Cardiopulmonary Bypass | | Mycobacterium chimaera | 2020 | doi:10.1016/j.jhin.2019.10.009 | International Society for Cardiovascular Infectious Diseases (ISCVID) | European Society of Cardiology (ESC) grading system | European Society of Cardiology (ESC) grading system | yes |
| 44 | Invasive Aspergillosis in solid-organ transplant recipients: Guidelines from the American Society of Transplantation Infectious Diseases Community of Practice | | Aspergillosis | 2019 | doi:10.1111/ctr.13544 | American Society of Transplantation (ASL) | unclear | unclear | unclear* |
| 45 | KDIGO 2022 Clinical Practice Guideline FOR the Prevention, Diagnosis, Evaluation, and Treatment of Hepatitis C in Chronic Kidney Disease | | Hepatitis C | 2022 | doi:10.1016/j.kint.2022.07.013 | Kidney Disease Improving Global Outcomes (KDIGO) | GRADE | GRADE | no |
| 46 | Management of intra-abdominal-infections: 2017 World Society of Emergency Surgery guidelines summary focused on remote areas and low-income nations | | Intra-abdominal-infections | 2020 | doi:10.1016/j.ijid.2020.07.046 | World Society of Emergency Surgery | none | none | unclear* |
| 47 | Middle-East OBGYN Graduate Education (MOGGE) Foundation practice guidelines: prevention of group B Streptococcus infection in pregnancy and in newborn. Practice guideline no. 02-O-20 | | Group B streptococcus infection | 2022 | doi:10.1080/14767058.2021.1875211 | Middle-East OBGYN Graduate Education (MOGGE) Foundation | OCEBM | OCEBM | yes |
| 48 | Mycobacterial infections in adults with haematological malignancies and haematopoietic stem cell transplants: guidelines from the 8th European Conference on Infections in Leukaemia | | Mycobacterial infections | 2022 | doi:10.1016/s1473-3099(22)00227-4 | European Conference on Infections in Leukaemia (ECIL) | IDSA-USPHS | Modified IDSA-USPHS | yes |
| 49 | Mycobacterium tuberculosis infections in solid organ transplantation: Guidelines from the infectious diseases community of practice of the American Society of Transplantation | | Mycobacterium tuberculosis | 2019 | doi:10.1111/ctr.13513 | American Society of Transplantation (ASL) | unclear | unclear | unclear* |
| 50 | Paediatric European Network for Treatment of AIDS (PENTA) guidelines for treatment of paediatric HIV-1 infection 2015: optimizing health in preparation for adult life | | AIDS | 2018 | doi:10.1111/hiv.12217 | Paediatric European Network for Treatment of AIDS (PENTA) | none | none | yes |
| 51 | Pneumocystis jiroveci in solid organ transplantation: Guidelines from the American Society of Transplantation Infectious Diseases Community of Practice | | Pneumocystis jiroveci | 2019 | doi:10.1111/ctr.13587 | American Society of Transplantation (ASL) | unclear | unclear | unclear* |
| 52 | Pneumonia in solid organ transplantation: Guidelines from the American Society of Transplantation Infectious Diseases Community of Practice | | Pneumonia | 2019 | doi:10.1111/ctr.13545 | American Society of Transplantation (ASL) | unclear | unclear | unclear* |
| 53 | Surviving Sepsis Campaign Guidelines on the Management of Adults With Coronavirus Disease 2019 (COVID-19) in the ICU: First Update | | COVID-19 | 2021 | doi:10.1097/ccm.0000000000004899 | Surviving Sepsis Campaign (SSC) | GRADE | GRADE | no |
| 54 | Surviving Sepsis Campaign International Guidelines for the Management of Septic Shock and Sepsis-Associated Organ Dysfunction in Children | | Septic Shock | 2020 | doi:10.1097/pcc.0000000000002198 | Surviving Sepsis Campaign (SSC) | GRADE | GRADE | yes |
| 55 | Treatment of Drug-Resistant Tuberculosis. An Official ATS/CDC/ERS/IDSA Clinical Practice Guideline | | Tuberculosis | 2019 | doi:10.1164/rccm.201909-1874ST | The American Thoracic Society (ATS), U.S. Centers for Disease Control and Prevention (CDC), European Respiratory Society (ERS), Infectious Diseases Society of America (IDSA) | GRADE | GRADE | no |
| 56 | Treatment of Nontuberculous Mycobacterial Pulmonary Disease: An Official ATS/ERS/ESCMID/IDSA Clinical Practice Guideline | | Nontuberculous Mycobacterial Pulmonary Disease | 2020 | doi:10.1093/cid/ciaa1125 | American Thoracic Society (ATS), European Respiratory Society (ERS), European Society of Clinical Microbiology and Infectious Diseases (ESCMID), and Infectious Diseases Society of America (IDSA) | GRADE | GRADE | yes |
| 57 | Update June 2022: management of hospitalised adults with coronavirus disease 2019 (COVID-19): a European Respiratory Society living guideline | | COVID-19 | 2022 | doi:10.1183/13993003.00803-2022 | European Respiratory Society (ERS) | GRADE | GRADE | no |
| 58 | Update of the EAU/ESPU guidelines on urinary tract infections in children | | Urinary tract infections | 2021 | doi:10.1016/j.jpurol.2021.01.037 | European Association of Urology (EAU), The European Society for Paediatric Urology (ESPU) | AHRQ | Modified GRADE | yes |
| 59 | Update to living WHO guideline on drugs to prevent covid-19 | | COVID-19 | 2023 | doi:10.1136/bmj.p692 | World Health Organization (WHO) | GRADE | GRADE | no |
| 60 | Updates to Recurrent Uncomplicated Urinary Tract Infections in Women: AUA/CUA/SUFU Guideline | | Uncomplicated Urinary Tract Infections | 2022 | doi:10.1097/ju.0000000000002860 | American Urological Association (AUA), Canadian Urological Association (CUA), Society of Urodynamics, Female Pelvic Medicine & Urogenital Reconstruction (SUFU) | AUA | AUA | yes |
| 61 | Urinary tract infections in solid organ transplant recipients: Guidelines from the American Society of Transplantation Infectious Diseases Community of Practice | | Urinary tract infections | 2019 | doi:10.1111/ctr.13507 | American Society of Transplantation (ASL) | GRADE | GRADE | no |
| 62 | Varicella zoster virus in solid organ transplantation: Guidelines from the American Society of Transplantation Infectious Diseases Community of Practice | | Varicella zoster | 2019 | doi:10.1111/ctr.13622 | American Society of Transplantation (ASL) | unclear | unclear | unclear* |
| 63 | Viral hepatitis: Guidelines by the American Society of Transplantation Infectious Disease Community of Practice | | Viral hepatitis | 2019 | doi:10.1111/ctr.13514 | American Society of Transplantation (ASL) | unclear | unclear | unclear* |
| 64 | WHO consolidated guidelines on tuberculosis: module 3: diagnosis: tests for TB infection | | Tuberculosis | 2022 | doi: | World Health Organization (WHO) | GRADE | GRADE | no |
| 65 | WHO guideline for the treatment of visceral leishmaniasis in HIV co-infected patients in East Africa and South-East Asia | | Visceral leishmaniasis | 2022 | doi:https://doi.org/10.1136/bmj.i2016 | World Health Organization (WHO) | GRADE | GRADE | no |
| 66 | WHO Therapeutics and COVID-19: living guideline | | COVID-19 | 2023 | Website | World Health Organization (WHO) | GRADE | GRADE | no |
|  | | *GRADE: Grading of Recommendations Assessment, Development, and Evaluation*  *IDSA-USPHS: Infectious Diseases Society of America and the United States Public Health Service*  *OCEBM: Oxford Centre for Evidence-Based Medicine*  *AHRQ: Agency for Healthcare Research and Quality's*  *AUA: American Urological Association*  *ACCF/AHA: American College of Cardiology and American Heart Association*  *ESC: European Society of Cardiology grading system* | | | | | | | |

*Not enough information to assess whether EO was used or not.

## S2 Table: Type of systems used for assessing quality of evidence in guidelines reporting use of a system.

|  | **Number of guidelines (n)**  **Total=66** | **Percentage (%)** |
| --- | --- | --- |
| **Reported systems for assessing the quality of evidence** | | |
| Grading of Recommendations Assessment, Development, and Evaluation (GRADE) | 29 | 43.9% |
| Infectious Diseases Society of America and the United States Public Health Service (IDSA-USPHS) | 10 | 15.1% |
| Modified from GRADE | 7 | 10.6% |
| Oxford Centre for Evidence-Based Medicine (OCEBM) | 2 | 3.0% |
| Agency for Healthcare Research and Quality's (AHRQ) | 2 | 3.0% |
| European Society of Cardiology (ESC) grading system | 2 | 3.0% |
| American Urological Association (AUA) | 1 | 1.5% |
| Unclear | 9 | 13.6% |
| No system | 4 | 6.0% |
| **Reported systems for rating the strength of recommendations** | | |
| Grading of Recommendations Assessment, Development, and Evaluation (GRADE) | 28 | 42.4% |
| Modified from GRADE | 10 | 15.1% |
| Modified from Infectious Diseases Society of America and the United States Public Health Service (IDSA-USPHS) | 6 | 9.1% |
| Infectious Diseases Society of America and the United States Public Health Service (IDSA-USPHS) | 3 | 4.5% |
| Oxford Centre for Evidence-Based Medicine (OCEBM) | 2 | 3.0% |
| European Society of Cardiology (ESC) grading system | 2 | 3.0% |
| Agency for Healthcare Research and Quality's (AHRQ) | 1 | 1.5% |
| American Urological Association (AUA) | 1 | 1.5% |
| Unclear | 9 | 13.6% |
| No system* | 4 | 6.0% |

*Not possible to identify the used system. All of these guidelines were published by American Society of Transplantation (ASL)

## S3 Table: Terms used for EO

|  | **Number of guidelines**  **Total=32** | **Percentage (%)** |
| --- | --- | --- |
| **Prevalence of terms used for expert opinion in guidelines allowing EO** | | |
| Expert opinion | 11 | 34.4% |
| Opinions of respected authorities | 12 | 37.5% |
| Expert judgement | 4 | 12.5% |
| Consensus of expert opinion | 2 | 6.2% |
| Consensus recommendation | 1 | 3.1% |
| Good practice principal | 1 | 3.1% |
| In our practice statement | 1 | 3.1% |

## S4 Table: List of verbs used for phrasing EO recommendations.

| **Verb** | **Number** | **%** |
| --- | --- | --- |
| should/should not | 130 | 34.4% |
| recommend/not recommend | 74 | 19.6% |
| consider | 38 | 10% |
| other (specific) verbs | 24 | 6.3% |
| may/might | 18 | 4.8% |
| is/are | 12 | 3.2% |
| indicated | 7 | 1.8% |
| suggest | 6 | 1.6% |
| use | 6 | 1.6% |
| can | 5 | 1.3% |
| review | 5 | 1.3% |
| must | 4 | 1.0% |
| required | 4 | 1.0% |
| include | 4 | 1.0% |
| need | 3 | 0.8% |
| provide | 3 | 0.8% |
| obtain | 3 | 0.8% |
| offer | 3 | 0.8% |
| target | 3 | 0.8% |
| give | 2 | 0.5% |
| advise | 2 | 0.5% |
| necessary | 1 | 0.3% |
| support | 1 | 0.3% |
| carry out | 1 | 0.3% |
| be alert | 1 | 0.3% |
| share | 1 | 0.3% |
| ensure | 1 | 0.3% |
| preferred | 1 | 0.3% |
| resulted | 1 | 0.3% |
| relieve | 1 | 0.3% |
| perform | 1 | 0.3% |
| send | 1 | 0.3% |
| start | 1 | 0.3% |
| manage | 1 | 0.3% |
| adhere | 1 | 0.3% |
| propose | 1 | 0.3% |
| screen | 1 | 0.3% |
| treat | 1 | 0.3% |
| measure | 1 | 0.3% |
| select | 1 | 0.3% |
| administer | 1 | 0.3% |
| intubate | 1 | 0.3% |
| acceptable | 1 | 0.3% |
| **Total** | **378** | **100%** |

## S5 Table: Publishing and endorsing organizations of included guidelines.

| **No.** | **Publishing and endorsing societies** |
| --- | --- |
| 1 | American Academy of Neurology (AAN) |
| 2 | American College of Rheumatology (ACR) |
| 3 | American Society for Microbiology (ASM) |
| 4 | American Society of Transplantation (ASL) |
| 5 | American Society of Tropical Medicine and Hygiene (ASTMH) |
| 6 | American Thoracic Society (ATS) |
| 7 | American Urological Association (AUA) |
| 8 | Arbeitsgemeinschaft Infektionen in der Hämatologie und Onkologie (AGIHO) |
| 9 | Asia Fungal Working Group (AFWG) |
| 10 | Asian Pacific association for the study of liver (APASL) |
| 11 | Asociación Argentina de Microbiología (AAM) |
| 12 | Asociacion Colombiana de Infectologia (ACIN) |
| 13 | Asociación Española de Micología (AEM) |
| 14 | Asociacíon Iberoamericana de Micologia Médica |
| 15 | Asociación Panamericana de Infectología (API) |
| 16 | Association Infection Prevention Control Nurse Indonesia |
| 17 | Association of Medical Microbiology and Infectious Disease (AMMI) |
| 18 | Australasian Antifungal Guidelines Steering Committee |
| 19 | Australasian Society for Infectious Diseases (ASID) |
| 20 | Austrian Society for Medical Mycology (OEGMM) |
| 21 | Belgian Society of Human and Animal Mycology (BSHAM) |
| 22 | Brazilian Association of Hematology (ABHH) |
| 23 | Brazilian Society of Infectious Diseases (SBI) |
| 24 | Brazilian Society of Microbiology |
| 25 | British Infection Association (BIA) |
| 26 | British Society for Medical Mycology (BSMM) |
| 27 | Canadian Urological Association (CUA) |
| 28 | Chinese Preventive Medicine Association (CPMA) |
| 29 | Chinese Society for Infection Control Sector |
| 30 | Clinical Infectious Disease Society (CIDS) |
| 31 | Czech Society for Medical Microbiology (API) |
| 32 | Dermato-venereological Branch of the European Union of Medical Specialists (UEMS), |
| 33 | Deutsche Gesellschaft für Infektiologie (DGI) |
| 34 | Dutch Society for Medical Mycology (NVMY) |
| 35 | Euro-Asian Society for Infectious Diseases (EASID) |
| 36 | European Academy of Dermatology and Venereology (EADV) |
| 37 | European AIDS Clinical Society (EACS) |
| 38 | European Association for the Study of the Liver (EASL) |
| 39 | European Association of Nuclear Medicine (EANM) |
| 40 | European Association of Urology (EAU) |
| 41 | European Confederation for Medical Mycology (ECMM) |
| 42 | European Conference on Infections in Leukaemia (ECIL) |
| 43 | European Dermatology Forum (EDF) |
| 44 | European Group for Blood and Marrow Transplantation (EBMT) |
| 45 | European Leukemia Net (ELN) |
| 46 | European Pediatric Mycology Network (EPMyN) |
| 47 | European Respiratory Society (ERS) |
| 48 | European Society for Vascular Surgery (ESVS) |
| 49 | European Society of Clinical Microbiology and Infectious Diseases (ESCMID) |
| 50 | European society of intensive care medicine (ESICM) |
| 51 | Federation of Infectious Diseases Societies of Southern Africa (FIDSSA) |
| 52 | Federazione Italiana di Micopatologia Umana e Animale (FIMUA) |
| 53 | Finnish Society for Medical Mycology (FSMM) |
| 54 | French Society for Medical Mycology (SFMM) |
| 55 | German Center for Infection Research (DZIF) |
| 56 | German-Speaking Mycological Society (DMykG) |
| 57 | Ghana Medical Mycology Group |
| 58 | Groupe de Recherche sur les Infections de Prothèses |
| 59 | Hellenic Society of Medical Mycology (HSMM) |
| 60 | Ho Chi Minh City Infection Control Society (HICS) |
| 61 | Hong Kong Infection Control Nurses Association (HKICNA) |
| 62 | Hungarian Society for Infectology and Clinical Microbiology (HUMMT) |
| 63 | Indian Society of Medical Mycologists (ISMM) |
| 64 | Indonesia Society for Human and Animal Mycology |
| 65 | Indonesia Society for Medical Mycology (IDAT) |
| 66 | Indonesian Society of Infection Control (INASIC) |
| 67 | Infection Control Association of Singaport (ICAS) |
| 68 | Infection Control Society of Taiwan (ICST) |
| 69 | Infectious Diseases and Clinical Microbiology Speciality Society of Turkey (EKMUD) |
| 70 | Infectious Diseases Society of America (IDSA) |
| 71 | Infectious Diseases Society of Taiwan (IDST) |
| 72 | Infectious Diseases Society of Thailand |
| 73 | International Immunocompromised Host Society (ICHS) |
| 74 | International Pediatric Fungal Network (IPPFN) |
| 75 | International Society for Cardiovascular Infectious Diseases (ISCVID) |
| 76 | International Society for Human and Animal Mycology (ISHAM) |
| 77 | International Society of Antimicrobial Chemotherapy (ISAC) |
| 78 | International Union Against Sexually Transmitted Infections (IUSTI) |
| 79 | Interregional Association for Clinical Microbiology and Antimicrobial Chemotherapy (IACMAC) |
| 80 | Iranian Society of Infectious Diseases and Tropical Medicine |
| 81 | Iranian Society of Medical Mycology (ISMM) |
| 82 | Irish Fungal Society (IFS) |
| 83 | Israel Society for Medical Mycology (ISMM) |
| 84 | Israeli Society of Infectious Diseases |
| 85 | Kidney Disease Improving Global Outcomes (KDIGO) |
| 86 | Korean Surgical Infection Society (KSIS) |
| 87 | Latin American Forum for Fungal Infections |
| 88 | Latin American Thoracic Association (ALAT) |
| 89 | Lebanese Society of Infectious Diseases and Clinical Microbiology (LSIDCM) |
| 90 | Malaysian Society of Infectious Diseases and Chemotherapy (MSIDC) |
| 91 | Medical Mycology and Infectious Diseases Society of Pakistan (MMIDSP) |
| 92 | Medical Mycology Society of Nigeria (MMSN) |
| 93 | Mexican Academy of Dermatology |
| 94 | Middle-East OBGYN Graduate Education (MOGGE) Foundation |
| 95 | Mycoses Study Group Education and Research Consortium (MSGERC) |
| 96 | National Institute for Health and Care Excellence (NICE) |
| 97 | National Nasocomial Infection Control Group Thailand |
| 98 | Nordic Society for Medical Mycology (NSMM) |
| 99 | Österreichische Gesellschaft für Infektionskrankheiten und Tropenmedizin (OEGIT) |
| 100 | Paediatric European Network for Treatment of AIDS (PENTA) |
| 101 | Paul-Ehrlich-Society for Chemotherapy |
| 102 | Pediatric Infectious Diseases Society (PIDS) |
| 103 | Peseutan Kawalan Infeksi dan Antimikrobial Kota Kinabalu Sabah (PKIAKKS) |
| 104 | Portuguese Association of Medical Mycology (ASPOMM) |
| 105 | Romanian Society of Medical Mycology and Mycotoxicology (RSMMM) |
| 106 | Royal College of General Practitioners (RCGP) |
| 107 | Scottish Intercollegiate Guidelines Network (SIGN) |
| 108 | Section for Mycology of Croatian Microbiological Society |
| 109 | Serbian Society of Medical Mycology |
| 110 | Slovak Society of Chemotherapy |
| 111 | Slovak Society of Infectious Diseases |
| 112 | Società Italiana Terapia Antinfettiva (SITA) |
| 113 | Society for Clinical Microbiologists of Turkey (KLIMUD) |
| 114 | Society for Healthcare Epidemiology of America (SHEA) |
| 115 | Society for Indian Human and Animal Mycology (ISMM ) |
| 116 | Society of Infectious Diseases Pharmacists (SIDP) |
| 117 | Society of Urodynamics, Female Pelvic Medicine & Urogenital Reconstruction (SUFU) |
| 118 | Sorveglianza Epidemiologica Infezioni nelle Emopatie (SEIFEM) |
| 119 | South African Society for Microbiology (SASM) |
| 120 | Spanish Society of Medical Microbiology and Infectious Diseases (SEIMC) |
| 121 | Study Group Education & Research Consortium (MSG ERC) |
| 122 | Surviving Sepsis Campaign (SSC) |
| 123 | Swedish Society for Clinical Mycology |
| 124 | Swiss Society of Microbiology (SSM) |
| 125 | Thai Medical Mycology Forum (TMMF) |
| 126 | The American Thoracic Society (ATS), |
| 127 | The Asia Pacific Society of Infection Control (APSIC) |
| 128 | the European Organization for Research and Treatment of Cancer (EORTC) |
| 129 | The European Society for Paediatric Urology (ESPU) |
| 130 | Turkish Febrile Neutropenia Society |
| 131 | Turkish Society for Clinical Microbiology and Infectious Diseases |
| 132 | Turkish Society of Hospital Infection and Control |
| 133 | Turkish Society of Medical Mycology |
| 134 | U.S. Centers for Disease Control and Prevention (CDC) |
| 135 | World Health Organization (WHO) |
| 136 | World Society of Emergency Surgery (WSES) |

## S6 Table: Excluded guidelines during full-text screening, with reasons.

| **No.** | **Title** | **Year** | **DOI/URL** | **Exclusion reason** |
| --- | --- | --- | --- | --- |
| 1 | 2017 European guideline for the management of pelvic inflammatory disease | 2018 | doi:10.1177/0956462417744099 | Not a society/organisation |
| 2 | 2017 European guideline for the screening, prevention and initial management of hepatitis B and C infections in sexual health settings | 2018 | doi:10.1177/0956462418767576 | Not a society/organisation |
| 3 | 2018 AAHA Infection Control, Prevention, and Biosecurity Guidelines | 2018 | doi:10.5326/jaaha-ms-6903 | Wrong population |
| 4 | 2018 European guideline on the organization of a consultation for sexually transmitted infections | 2019 | doi:10.1111/jdv.15577 | Wrong population |
| 5 | 2019 European guideline on the management of lymphogranuloma venereum | 2019 | doi:10.1111/jdv.15729 | Not a society/organisation |
| 6 | 2019 IUSTI-Europe guideline for the management of anogenital warts | 2020 | doi:10.1111/jdv.16522 | Wrong population |
| 7 | A rapid advice guideline for the diagnosis and treatment of 2019 novel coronavirus (2019-nCoV) infected pneumonia (standard version) | 2020 | doi: 10.1186/s40779-020-0233-6 | National guideline |
| 8 | AAOS Clinical Practice Guideline Summary: Prevention of Surgical Site Infection After Major Extremity Trauma | 2023 | doi:10.5435/jaaos-d-22-00792 | National guideline |
| 9 | AARC Clinical Practice Guideline: Management of Pediatric Patients With Oxygen in the Acute Care Setting | 2021 | doi:10.4187/respcare.09006 | National guideline |
| 10 | AAUS guideline for acute bacterial prostatitis 2021 | 2021 | doi:10.1016/j.jiac.2021.06.001 | panel not international |
| 11 | AAUS guidelines 2021 revision sexually transmitted infection (STIs) diagnostic strategy for STI | 2022 | doi:10.1016/j.jiac.2021.09.002 | Wrong publication type |
| 12 | ACG Clinical Guidelines: Prevention, Diagnosis, and Treatment of Clostridioides difficile Infections | 2021 | doi:10.14309/ajg.0000000000001278 | National guideline |
| 13 | Adult Outpatients With Acute Cough Due to Suspected Pneumonia or Influenza: CHEST Guideline and Expert Panel Report | 2019 | doi:10.1016/j.chest.2018.09.016 | National guideline |
| 14 | An evaluation of WHO emergency guidelines for Zika virus disease | 2019 | doi:10.1111/jebm.12347 | Wrong publication type |
| 15 | Antibiotic lock therapy for the conservative treatment of long-term intravenous catheter-related infections in adults and children: When and how to proceed? Guidelines for clinical practice 2020 | 2021 | doi:10.1016/j.idnow.2021.02.004 | National guideline |
| 16 | Antiretroviral treatment indications and adherence to the German-Austrian treatment initiation guidelines in the German ClinSurv HIV Cohort between 1999 and 2016 | 2019 | doi:10.1007/s15010-018-1248-8 | Wrong publication type |
| 17 | APSIC guidelines for disinfection and sterilization of instruments in health care facilities | 2018 | doi:10.1186/s13756-018-0308-2 | Wrong population |
| 18 | Arenaviruses and West Nile Virus in solid organ transplant recipients: Guidelines from the American Society of Transplantation Infectious Diseases Community of Practice | 2019 | doi:10.1111/ctr.13576 | Panel not international |
| 19 | ASGE guideline for infection control during GI endoscopy | 2018 | doi: 10.1016/j.gie.2017.12.009 | Wrong population |
| 20 | Asian guidelines for condyloma acuminatum | 2022 | doi:10.1016/j.jiac.2022.03.004 | Panel not international |
| 21 | Asian guidelines for genital herpes | 2021 | doi:10.1016/j.jiac.2021.07.015 | Panel not international |
| 22 | Asian guidelines for syphilis | 2022 | doi:10.1016/j.jiac.2022.04.023 | Panel not international |
| 23 | ATS/CDC/ERS/IDSA Clinical Practice Guidelines for Treatment of Drug-Resistant Tuberculosis: A Two-edged Sword? | 2020 | doi:10.1164/rccm.201912-2460LE | Wrong publication type |
| 24 | CATS Guideline for management of Children with SARS-CoV-2 infection (including PIMS-TS) | 2022 | https://cats.nhs.uk/wp-content/uploads/cats_SARS-CoV-2_PIMS_TS_2022.pdf | National guideline |
| 25 | Chagas disease: comments on the 2018 PAHO Guidelines for diagnosis and management | 2019 | doi:10.1093/jtm/taz060 | Wrong publication type |
| 26 | Clinical Practice Guideline by the Pediatric Infectious Diseases Society and the Infectious Diseases Society of America: 2021 Guideline on Diagnosis and Management of Acute Hematogenous Osteomyelitis in Pediatrics | 2021 | doi:10.1093/jpids/piab027 | Duplicate |
| 27 | Clinical Practice Guideline for Systemic Antifungal Prophylaxis in Pediatric Patients With Cancer and Hematopoietic Stem-Cell Transplantation Recipients | 2020 | doi:10.1200/jco.20.00158 | National guideline |
| 28 | Clinical Practice Guideline on the Diagnosis and Prevention of Periprosthetic Joint Infections | 2019 | https://www.aaos.org/contentassets/9a006edd608c468ba066624defca5502/pji-clinical-practice-guideline-final-9-18-19-.pdf | National guideline |
| 29 | Clinical practice guideline on the management of septic shock and sepsis-associated organ dysfunction in children: Endorsement by the Scandinavian Society of Anaesthesiology and Intensive Care Medicine | 2021 | doi:10.1111/aas.13958 | Wrong publication type |
| 30 | Clinical Practice Guidelines by the Infectious Diseases Society of America (IDSA), American Academy of Neurology (AAN), and American College of Rheumatology (ACR): 2020 Guidelines for the Prevention, Diagnosis, and Treatment of Lyme Disease | 2021 | doi:10.1002/acr.24495 | Duplicate |
| 31 | Clinical Practice Guidelines by the Infectious Diseases Society of America, American Academy of Neurology, and American College of Rheumatology: 2020 Guidelines for the Prevention, Diagnosis and Treatment of Lyme Disease | 2021 | doi:10.1212/wnl.0000000000011422 | Duplicate |
| 32 | Clinical Practice Guidelines for Clostridium difficile Infection in Adults and Children: 2017 Update by the Infectious Diseases Society of America (IDSA) and Society for Healthcare Epidemiology of America (SHEA) | 2018 | doi:10.1093/cid/ciy149 | Old version |
| 33 | Clostridioides difficile Infection: A Focused Guideline Update From the IDSA | 2022 | doi: | Wrong publication type |
| 34 | Consensus guidelines for antifungal stewardship, surveillance and infection prevention, 2021 | 2021 | doi:10.1111/imj.15586 | Wrong population |
| 35 | Consensus guidelines for the diagnosis and management of cryptococcosis and rare yeast infections in the haematology/oncology setting, 2021 | 2021 | doi:10.1111/imj.15590 | Not a society/organisation |
| 36 | Corticosteroid therapy for sepsis: a clinical practice guideline | 2018 | doi:10.1136/bmj.k3284 | Not a society/organisation |
| 37 | Diabetic foot infections antibiotic management clinical guideline | 2019 | https://www.sahealth.sa.gov.au/wps/wcm/connect/public+content/sa+health+internet/resources/policies/diabetic+foot+infections+antibiotic+management+clinical+guideline | National guideline |
| 38 | Editor's Choice - European Society for Vascular Surgery (ESVS) 2020 Clinical Practice Guidelines on the Management of Vascular Graft and Endograft Infections | 2020 | doi:10.1016/j.ejvs.2019.10.016 | Duplicate |
| 39 | ESCMID COVID-19 living guidelines: drug treatment and clinical management | 2022 | doi:10.1016/j.cmi.2021.11.007 | Adoption/adaptation |
| 40 | ESCMID guidelines on testing for SARS-CoV-2 in asymptomatic individuals to prevent transmission in the health care setting | 2022 | doi:10.1016/j.cmi.2022.01.007 | Wrong population |
| 41 | European Association of Urology Guidelines Office Rapid Reaction Group: An Organisation-wide Collaborative Effort to Adapt the European Association of Urology Guidelines Recommendations to the Coronavirus Disease 2019 Era | 2020 | doi:10.1016/j.eururo.2020.04.056 | Wrong population |
| 42 | European guidelines for primary antifungal prophylaxis in adult haematology patients: summary of the updated recommendations from the European Conference on Infections in Leukaemia | 2018 | doi:10.1093/jac/dky286 | Duplicate |
| 43 | European society of clinical microbiology and infectious diseases guidelines for coronavirus disease 2019: an update on treatment of patients with mild/moderate disease | 2022 | doi:10.1016/j.cmi.2022.08.013 | Adoption/adaptation |
| 44 | European Society of Clinical Microbiology and Infectious Diseases Study Group for Infections in Compromised Hosts: Guidelines for Infectious and Immunological Complications of Targeted and Biological Therapies | 2020 | doi:10.1016/j.jaip.2019.10.001 | Not a society/organisation |
| 45 | Evidence-based updates to the 2021 Surviving Sepsis Campaign guidelines Part 2: Guideline review and clinical application | 2022 | doi:10.1097/01.NPR.0000904452.61601.a5 | Duplicate |
| 46 | Executive Summary of JSMM Clinical Practice Guidelines for Diagnosis and Treatment of Cryptococcosis 2019 | 2020 | doi:10.3314/mmj.20.001 | National guideline |
| 47 | Group B Streptococcus Disease:​ AAP Updates Guidelines for the Management of At-Risk Infants | 2020 | https://www.aafp.org/content/dam/brand/aafp/pubs/afp/issues/2020/0315/p378.pdf | Not a society/organisation |
| 48 | Guideline for the Management of Clostridium Difficile Infection in Children and Adolescents With Cancer and Pediatric Hematopoietic Stem-Cell Transplantation Recipients | 2018 | doi:10.1200/jco.18.00407 | Not a society/organisation |
| 49 | Guideline Quick View: Transmission-Based Precautions | 2019 | doi:10.1002/aorn.12675 | Wrong publication type |
| 50 | Guidelines for Clostridium difficile infection in adults | 2020 | doi:10.5114/pg.2020.93629 | National guideline |
| 51 | Guidelines for COVID-19 Management in Hematopoietic Cell Transplantation and Cellular Therapy Recipients | 2020 | doi:10.1016/j.bbmt.2020.07.027 | National guideline |
| 52 | Guidelines for diagnosing 'long Covid' in patients living with postacute sequelae of COVID-19 (PASC) | 2023 | doi:10.1136/ebnurs-2022-103538 | Wrong publication type |
| 53 | Helicobacter pylori World Gastroenterology Organization Global Guideline | 2023 | doi:10.1097/mcg.0000000000001719 | Wrong publication type |
| 54 | IFCC Interim Guidelines on Molecular Testing of SARS-CoV-2 Infection | 2020 | doi:10.1515/cclm-2020-1412 | Wrong population |
| 55 | Infectious Diarrhea: IDSA Updates Guidelines for Diagnosis and Management | 2018 | https://www.aafp.org/content/dam/brand/aafp/pubs/afp/issues/2018/0515/p676.pdf | Wrong publication type |
| 56 | Infectious Diseases Society of America Guidelines on the Diagnosis of COVID-19 | 2020 | https://www.idsociety.org/globalassets/idsa/practice-guidelines/covid-19/diagnostics/idsa-covid-19-guideline_dx_version-1.0.1.pdf | Old version |
| 57 | Infectious Diseases Society of America Guidelines on the Diagnosis of COVID-19: Serologic Testing | 2020 | https://www.idsociety.org/practice-guideline/covid-19-guideline-serology/ | Old version |
| 58 | Infectious Diseases Society of America Guidelines on the Diagnosis of COVID-19:Serologic Testing | 2020 | doi:10.1093/cid/ciaa1343 | part of a same guideline |
| 59 | Infectious Diseases Society of America Guidelines on the Treatment and Management of Patients with COVID-19 | 2020 | https://www.idsociety.org/globalassets/idsa/practice-guidelines/covid-19/treatment/idsa-covid-19-gl-tx-and-mgmt-v3.5.1.pdf | Old version |
| 60 | Infectious Diseases Society of America Guidelines on the Treatment and Management of Patients with COVID-19 | 2021 | https://www.idsociety.org/globalassets/idsa/practice-guidelines/covid-19/treatment/idsa-covid-19-gl-tx-and-mgmt-v5.3.1.pdf | Old version |
| 61 | Infectious Diseases Society of America Guidelines on the Treatment and Management of Patients with COVID-19 | 2022 | doi:10.1093/cid/ciac724 | Old version |
| 62 | Inpatient Adult and Pediatric Clostridium difficile Infection Guideline | 2019 | https://michmed-public.policystat.com/policy/8071880/latest/ | National guideline |
| 63 | Interim infection prevention and control guidelines for the management of COVID-19 in healthcare settings | 2020 | https://www.health.qld.gov.au/__data/assets/pdf_file/0038/939656/qh-covid-19-Infection-control-guidelines.pdf | Wrong population |
| 64 | International Guidelines 2020 for the Management of Septic Shock in Children | 2020 | https://www.indianpediatrics.net/july2020/671.pdf | Not a society/organisation |
| 65 | JAID/JSC Guidelines for Infection Treatment 2015-Intestinal infections | 2018 | doi:10.1016/j.jiac.2017.09.002 | National guideline |
| 66 | Joint EANM-ESR-ESCMID-EBJIS guidelines for imaging prosthetic joint infection | 2019 | doi:10.1007/s00259-019-4263-9 | Wrong publication type |
| 67 | Joint Healthcare Infection Society (HIS) and Infection Prevention Society (IPS) guidelines for the prevention and control of meticillin-resistant Staphylococcus aureus (MRSA) in healthcare facilities | 2021 | doi:10.1016/j.jhin.2021.09.022 | Wrong population |
| 68 | KASL clinical practice guidelines for management of chronic hepatitis B | 2019 | doi:10.3350/cmh.2019.1002 | National guideline |
| 69 | Management of covid-19: a practical guideline for maternal and newborn health care providers in Sub-Saharan Africa | 2022 | doi:10.1080/14767058.2020.1763948 | Wrong population |
| 70 | Management of hospitalised adults with coronavirus disease 2019 (COVID-19): a European Respiratory Society living guideline | 2021 | doi:10.1183/13993003.00048-2021 | Old version |
| 71 | Management of Pediatric Lyme Disease: Updates From 2020 Lyme Guidelines | 2022 | doi:10.1542/peds.2021-054980 | Not a society/organisation |
| 72 | Managing COVID-19 symptoms (including at the end of life) in the community: summary of NICE guidelines | 2020 | doi:10.1136/bmj.m1461 | Wrong publication type |
| 73 | Mycobacterial infections in adults with haematological malignancies and haematopoietic stem cell transplants: guidelines from the 8th European Conference on Infections in Leukaemia | 2022 | doi:10.1016/S1473-3099(22)00227-4 | Duplicate |
| 74 | New guidelines for severe community-acquired pneumonia | 2021 | doi:10.1097/mcp.0000000000000760 | Wrong publication type |
| 75 | New Sepsis Guidelines Specific to Pediatrics | 2020 | doi:10.4037/ccn2020491 | Wrong publication type |
| 76 | NICE guideline on long COVID | 2021 | doi:10.1016/s2213-2600(21)00031-x | Wrong publication type |
| 77 | Paediatric European Network for Treatment of AIDS (PENTA) guidelines for treatment of paediatric HIV-1 infection 2015: optimizing health in preparation for adult life | 2018 | doi: 10.1111/hiv.12217 | Duplicate |
| 78 | Practice guidelines for the management of adult community-acquired urinary tract infections | 2018 | doi:10.1016/j.medmal.2018.03.005 | National guideline |
| 79 | Re: Recurrent Uncomplicated Urinary Tract Infections in Women: AUA/CUA/SUFU Guideline | 2019 | doi:10.1097/ju.0000000000000502 | Wrong publication type |
| 80 | Recurrent Uncomplicated Urinary Tract Infections in Women: AUA/CUA/SUFU Guideline | 2019 | 10.1097/JU.0000000000000296 | Old version |
| 81 | Sepsis Guidelines | 2019 | doi:10.1056/NEJMclde1815472 | Wrong publication type |
| 82 | South Asian Transplant Infectious Disease Guidelines for Solid Organ Transplant Candidates, Recipients, and Donors | 2023 | doi:10.1097/tp.0000000000004521 | Not a society/organisation |
| 83 | Surgical Infection Society 2020 Updated Guidelines on the Management of Complicated Skin and Soft Tissue Infections | 2021 | doi:10.1089/sur.2020.436 | National guideline |
| 84 | Surgical Infection Society Guidelines for Antibiotic Use in Patients Undergoing Cholecystectomy for Gallbladder Disease | 2022 | doi:10.1089/sur.2021.207 | National guideline |
| 85 | Surgical Infection Society Guidelines: 2022 Updated Guidelines for Antibiotic Use in Open Extremity Fractures | 2022 | doi:10.1089/sur.2022.206 | National guideline |
| 86 | Surviving Sepsis Campaign International Guidelines | 2020 | doi:10.1542/peds.2020-0629 | Wrong publication type |
| 87 | Surviving sepsis campaign international guidelines for the management of septic shock and sepsis-associated organ dysfunction in children | 2020 | doi:10.1007/s00134-019-05878-6 | Duplicate |
| 88 | Surviving Sepsis Campaign: Guidelines on the Management of Critically Ill Adults with Coronavirus Disease 2019 (COVID-19) | 2020 | doi:10.1097/ccm.0000000000004363 | Old version |
| 89 | Surviving Sepsis Campaign: guidelines on the management of critically ill adults with Coronavirus Disease 2019 (COVID-19) | 2020 | doi:10.1007/s00134-020-06022-5 | Duplicate |
| 90 | Surviving sepsis campaign: International guidelines for management of sepsis and septic shock in adults 2021 - endorsement by the Scandinavian society of anaesthesiology and intensive care medicine | 2022 | doi:10.1111/aas.14045 | Wrong publication type |
| 91 | The American Society of Colon and Rectal Surgeons Clinical Practice Guidelines for the Management of Anorectal Abscess, Fistula-in-Ano, and Rectovaginal Fistula | 2022 | doi:10.1097/dcr.0000000000002473 | National guideline |
| 92 | The EAU and AUA/CUA/SUFU Guidelines on Recurrent Urinary Tract Infections: What is the Difference? | 2020 | doi:10.1016/j.eururo.2020.06.032 | Wrong publication type |
| 93 | The Infectious Diseases Society of America Guidelines on the Diagnosis of COVID-19: Antigen Testing | 2021 | doi:10.1093/cid/ciab557 | Old version |
| 94 | The Infectious Diseases Society of America Guidelines on the Diagnosis of COVID-19: Antigen Testing | 2023 | doi:10.1093/cid/ciad032 | part of a same guideline |
| 95 | The Infectious Diseases Society of America Guidelines on the Diagnosis of COVID-19: Molecular Diagnostic Testing | 2020 | https://www.idsociety.org/globalassets/idsa/practice-guidelines/covid-19/diagnostics/idsa-covid-19-gl-dx-v2.0.0.pdf | Old version |
| 96 | The Infectious Diseases Society of America Guidelines on the Diagnosis of COVID-19: Molecular Diagnostic Testing | 2021 | doi:10.1093/cid/ciab048 | part of a same guideline |
| 97 | The JAID/JSC guidelines for management of infectious diseases 2017 - Sepsis and catheter-related bloodstream infection | 2021 | doi:10.1016/j.jiac.2019.11.011 | National guideline |
| 98 | Tissue and blood protozoa including toxoplasmosis, Chagas disease, leishmaniasis, Babesia, Acanthamoeba, Balamuthia, and Naegleria in solid organ transplant recipients- Guidelines from the American Society of Transplantation Infectious Diseases Community of Practice | 2019 | doi:10.1111/ctr.13546 | National guideline |
| 99 | Treatment of nontuberculous mycobacterial pulmonary disease: an official ATS/ERS/ESCMID/IDSA clinical practice guideline | 2020 | doi:10.1183/13993003.00535-2020 | Duplicate |
| 100 | Update on Clinical Practice Guidelines for Human Immunodeficiency Virus | 2020 | doi:10.1016/j.cnur.2020.06.005 | Wrong publication type |
| 101 | Update on Guidelines for Sexually Transmitted Infection Treatment and Management in the Adult and Adolescent Population | 2020 | doi:10.1016/j.cnur.2020.06.001 | Not a society/organisation |
| 102 | Update to living WHO guideline on drugs for covid-19 | 2020 | doi:10.1136/bmj.m4779 | Old version |
| 103 | Update to living WHO guideline on drugs for covid-19 | 2022 | doi:10.1136/bmj.o1713 | Duplicate |
| 104 | Updates to Recurrent Uncomplicated Urinary Tract Infections in Women: AUA/CUA/SUFU Guideline | 2022 | doi:10.1097/ju.0000000000002888 | Duplicate |
| 105 | Updating Your Practice: The 2017 Sepsis Guidelines | 2019 | doi:10.4037/ccn2019496 | Wrong publication type |
| 106 | Vaccination of solid organ transplant candidates and recipients: Guidelines from the American society of transplantation infectious diseases community of practice | 2019 | doi:10.1111/ctr.13563 | Wrong population |
| 107 | What is new in BTS 2017 & ATS/ERS/ESCMID/IDSA 2020 guidelines on treatment of non-tuberculous mycobacterial pulmonary disease? | 2021 | doi:10.4103/ijmr.ijmr_2573_21 | Wrong publication type |
| 108 | WHO COVID-19 therapeutic guidelines | 2021 | doi:10.1016/s0140-6736(21)01331-3 | Wrong publication type |
| 109 | WHO Guidelines for Prevention, Care and Treatment of Individuals Infected with HBV: A US Perspective | 2019 | doi:10.1016/j.cld.2019.04.008 | Wrong publication type |
| 110 | World Health Organization 2018 treatment guidelines for rifampicin-resistant tuberculosis: uncertainty, potential risks and the way forward | 2020 | doi:10.1016/j.ijantimicag.2019.10.003 | Wrong publication type |
| 111 | World Health Organization Guidelines on Treatment of Hepatitis C Virus Infection: Best Practice Advice From the American College of Physicians | 2020 | doi:10.7326/M19-3860 | Wrong publication type |
| 112 | World Society of Emergency Surgery-American Association for the Surgery of Trauma Guidelines for management of Clostridioides (Clostridium) difficile infection in surgical patients: An executive summary | 2021 | doi:10.1097/ta.0000000000003196 | Duplicate |
